# Association of a multiple risk factor assessment and intervention program with risk of diabetes: A population-based matched cohort study

**DOI:** 10.1371/journal.pmed.1005043

**Published:** 2026-09-21

**Authors:** Ernst Bergman, Anna Nordström, Lars Nyberg, Peter Nordström

**Affiliations:** 1 Department of Public Health and Caring Sciences, Clinical Geriatrics, Uppsala University, Uppsala, Sweden; 2 Department of Medical Sciences, Rehabilitation Medicine, Uppsala University, Uppsala, Sweden; 3 School of Sport Sciences, UiT, The Arctic University of Norway, Tromsø, Norway; 4 Department of Diagnostics and Intervention, Umeå Center for Functional Brain Imaging, Umeå University, Umeå, Sweden; 5 Department of Medical and Translational Biology, Umeå University, Umeå, Sweden; South African Medical Research Council, SOUTH AFRICA

## Abstract

**Background:**

Type 2 diabetes incidence remains high among older adults and contributes substantially to morbidity and healthcare burden. Most evidence supporting diabetes prevention derives from intensive lifestyle interventions targeting high-risk individuals under controlled trial conditions. The effectiveness of low-intensity, population-based prevention programs embedded in routine care remains uncertain. We evaluated whether participation in a comprehensive health assessment and counseling program was associated with reduced incidence of diabetes in older adults.

**Methods and findings:**

We conducted a population-based matched cohort study including 70-year-old residents of Umeå municipality, Sweden, enrolled between 2012 and 2022 and followed through December 2022. The intervention consisted of a comprehensive health assessment followed by individualized motivational counseling, without structured long-term follow-up. Participants were matched 1:10 to controls from the general Swedish population by birth year, sex, and educational level. Individuals with prevalent diabetes were excluded. The primary outcome was incident diabetes, defined as a first recorded diagnosis in specialist care and/or first dispensed glucose-lowering medication, identified through nationwide health and prescription registers.

In total, 6,018 participants and 57,543 matched controls were included. During a mean follow-up of 4.9 years (standard deviation 2.9) in the intervention cohort and 4.8 years (standard deviation 2.9) in the control cohort, diabetes occurred in 335 (5.6%) participants and 3,919 (6.8%) controls. Participation was associated with a lower risk of incident diabetes (adjusted hazard ratio 0.76; 95% confidence interval [0.68,0.85]; *p* < 0.001). The association remained stable over time. The absolute risk reduction was 1.46 percentage points (95% confidence interval [0.91,1.98]; *p* < 0.001) at 5 years and 3.42 percentage points (95% confidence interval [2.13,4.75]; *p* < 0.001) at 10 years. Results were broadly consistent across the examined subgroups. The observational study design limits certainty regarding the intervention’s role in the observed association, and voluntary participation in the program may have introduced selection bias, healthy-volunteer effect, and residual confounding.

**Conclusions:**

In this population-based cohort study, participation in a low-intensity preventive health program delivered in routine care was associated with a lower incidence of diabetes among older adults. However, the observational study design limits evidence on the intervention’s role in the observed association, and more robust evidence is needed. Nevertheless, these findings suggest that scalable, population-wide prevention strategies may complement intensive high-risk approaches in addressing diabetes in aging populations.

## Author summary

**Why was this study done?:**

- Type 2 diabetes is common in older adults and contributes substantially to morbidity and healthcare costs.
- Most evidence for diabetes prevention comes from intensive lifestyle programs targeting selected high-risk individuals in clinical trials.
- It remains unclear whether low-intensity, population-based prevention programs delivered in routine healthcare settings can reduce diabetes risk in the general older population.

**What did the researchers do and find?:**

- We conducted a population-based matched cohort study including 6,018 participants in a preventive health assessment and counseling program and 57,543 matched controls.
- Participants were 70-year-old residents of Umeå municipality, Sweden, free from diabetes at baseline.
- During a mean follow-up of 4.8 years, participation in the program was associated with a 24% lower risk of developing diabetes.
- The absolute difference in diabetes risk between groups increased over time and was consistent across most examined subgroups.

**What do these findings mean?:**

- In this real-world setting, participation in a low-intensity preventive health program was associated with lower diabetes incidence among older adults.
- The findings suggest that scalable prevention strategies embedded in routine care may contribute to reducing diabetes burden at the population level, where even modest reductions in individual risk could translate into substantial public health benefits.
- The observational study design does not allow causal conclusions, and voluntary participation may have resulted in a healthier group of participants than non-participants.

## Abstract 3

In this population-based study, Ernst Bergman and colleagues found that older adults who participated in a health assessment followed by a personalized counselling session were less likely to develop type 2 diabetes than matched non-participants. These results suggest that a low-intensity intervention embedded in primary care may help support diabetes prevention in ageing populations.

## Introduction

Diabetes mellitus is a major public health challenge and is highly prevalent among older adults [1]. It contributes substantially to cardiovascular morbidity, functional decline, and healthcare utilization worldwide [2–4]. In Sweden and many other countries [5], diabetes incidence increases with age before plateauing in the oldest age groups [6]. Preventing diabetes in aging populations is therefore an important clinical and public health priority.

Strong evidence supports lifestyle modification for diabetes prevention, primarily from randomized controlled trials targeting individuals at elevated risk through intensive, structured programs [7–11]. Although these interventions have demonstrated substantial reductions in diabetes incidence under controlled conditions, their intensity, resource requirements, and selective inclusion criteria may limit scalability and sustainability in routine healthcare setting [12–14]. Whether lower-intensity, population-based prevention strategies embedded in routine care can influence diabetes incidence in the general older population remains uncertain.

Population-wide preventive initiatives implemented in European and Nordic settings have reported improvements in lifestyle behaviors and cardiometabolic risk profiles [15–18]. However, evidence regarding their association with hard clinical outcomes such as incident diabetes is limited, and older adults are frequently underrepresented [15]. Moreover, little is known about whether potential associations are consistent across demographic and clinical subgroups in real-world healthcare contexts [19].

The Healthy Ageing Initiative (HAI) is a population-wide preventive health program offered to all 70-year-olds within a defined Swedish healthcare region [20]. The program includes a comprehensive health assessment followed by individualized motivational counseling delivered within routine care, without structured long-term follow-up. Previous analyses have shown that participation in this program was associated with reduced cardiovascular disease (CVD) incidence [21]. Whether participation is also associated with reduced diabetes incidence has not been evaluated. Using a population-based matched cohort design with nationwide register follow-up, we examined whether participation in this low-intensity, routine-care prevention program was associated with incident diabetes among older adults. We also assessed whether any observed association was consistent across key baseline characteristics.

## Methods

### Study design and setting

We conducted a population-based matched cohort study examining the association between participation in the HAI, a primary prevention program in Umeå municipality, Sweden [20], and incident diabetes. Participants in the program were matched to controls from the general Swedish population. Follow-up was performed through linkage to nationwide registers, including the National Patient Register, the Prescribed Drug Register, and the Cause of Death Register [22–24], which provide near-complete national coverage of diagnoses, dispensed medications, and mortality. The study period spanned from June 1, 2012, to December 31, 2022. The study was conducted and reported in accordance with Strengthening the Reporting of Observational Studies in Epidemiology (STROBE) guidelines (S1 Checklist).

#### Healthy Ageing Initiative cohort.

The intervention cohort comprised 54% of 70-year-old residents invited within the municipality during the study period. Participation was open to all eligible individuals, with no exclusion criteria applied. The program included two structured health assessment visits conducted within routine care, during which lifestyle factors and cardiometabolic risk markers were evaluated. It was designed as a pragmatic, low-intensity program that could be implemented in routine clinical practice to target the major modifiable risk factors for CVD and diabetes at a population level. Participants received individualized feedback and motivational counseling aimed at promoting lifestyle changes relevant to cardiovascular and metabolic disease prevention. No structured long-term follow-up was provided within the program, and participants subsequently returned to ordinary care. Individuals with findings suggestive of undiagnosed conditions were advised to consult their general practitioner. A detailed description of the program is provided in S1 Appendix, and similar descriptions have previously been published [21,25]. The participants included in the program were generally healthier, with a lower prevalence of CVD and diabetes, than Umeå residents who did not participate [21].

#### Control cohort.

A control cohort was drawn from the general Swedish population by Statistics Sweden and individually matched 1:10 to HAI participants exactly on birth year, sex, and educational level [26]. Controls were required to be alive and free of diabetes at the index date corresponding to their matched participant and were assigned the same index date to ensure comparable follow-up time.

#### Exclusion criteria.

Individuals in both cohorts were excluded if they had prevalent diabetes at baseline, defined as a recorded diagnosis of diabetes (International Statistical Classification of Diseases and Related Health Problems, 10th Revision, ICD-10 codes E10 and E11) in the National Patient Register or a dispensed prescription of glucose-lowering medication (Anatomical Therapeutic Chemical Classification System, ATC code A10) before or on the index date. Individuals with missing data on matching variables were also excluded.

### Outcome definition

The primary outcome was incident diabetes during follow-up. Incident cases were identified through nationwide register linkage to the National Patient Register and the Prescribed Drug Register. Diabetes was defined as a first recorded diagnosis in specialized inpatient or outpatient care (ICD-10 code E11) or a first dispensed prescription of glucose-lowering medication (ATC code A10), whichever occurred first. The date of the first qualifying diagnosis or prescription was used as the event date. Participants were censored at death, identified through the Cause of Death Register, or at the end of follow-up.

### Covariates

Baseline covariates were specified prior to data analysis, based on subject-matter knowledge, and the availability of consistently recorded variables in the nationwide registers. Age, sex, and educational level were accounted for through matching. Additional covariates were selected to capture baseline differences in cardiometabolic morbidity, overall health and functional status, and medication use that could confound the eventual association with intervention and diabetes risk. Country of birth (born in Sweden versus elsewhere) was used as a proxy for broader sociodemographic health-related differences. Home care services were included as an indicator of functional dependency. Pre-existing diagnoses, identified using ICD-10 codes, included myocardial infarction (I21), stroke (I61, I63, I64), angina pectoris (I20), renal disease (N17-N19), chronic obstructive pulmonary disease (J44), cancer of the colon and rectum (C18, C20), breast cancer (C50), mental and behavioral disorders due to alcohol use (F10). Medication covariates, identified using ATC codes, included antihypertensive medications (C03, C07-C09), lipid-lowering agents (C10), antidepressants (N06A), and prednisolone (H02AB06). All covariates were ascertained on or before the index date. No data-driven variable selection procedures were applied.

### Statistical analysis

Follow-up time was calculated from the index date (defined as the second visit within the HAI program for participants and the corresponding assigned date for controls) until the first occurrence of incident diabetes, death, or end of follow-up (December 31, 2022), whichever occurred first. Descriptive statistics were used to summarize baseline characteristics.

The unadjusted association between participation in the HAI program and incident diabetes was estimated using Cox proportional hazards regression models stratified by matched set to account for individual matching. The proportional hazards assumption was assessed using Schoenfeld residuals. Adjusted hazard ratios (HRs) and cumulative incidence functions were estimated using Royston–Parmar flexible parametric survival models (with splines at the 25th, 50th and 75th percentiles of uncensored log survival times) with robust standard errors clustered by matched set. Royston–Parmar models provide smooth estimates of the underlying survival function, allowing direct estimation of adjusted cumulative risks and absolute risk differences. The Royston–Parmar models can also accommodate potential violations of the proportional hazards assumption (i.e., time-varying intervention effects). Absolute risk estimates and confidence intervals (CIs) were obtained through refitted bootstrap resampling. Competing-risk sensitivity analyses were performed using Fine–Gray subdistribution hazard models treating death as a competing event to evaluate the robustness of the observed association and to estimate the cumulative incidence of diabetes.

Effect modification was assessed by including interaction terms between intervention status and predefined baseline characteristics. Population attributable fractions (PAF) were estimated using model-based predictions to quantify the proportion of incident diabetes statistically attributable to selected baseline risk factors, assuming causal associations.

A general research plan, including the planned analytical approach, was agreed upon within the research team before data analysis, although it was not formally documented. The analyses reported were planned before data analysis, and no additional data-driven analyses were performed. Statistical analyses and creation of figures were performed in R (version 4.5.2; R Foundation for Statistical Computing, Vienna, Austria) in RStudio (version 2025.09.2+418; Posit Software, PBC, Boston, MA, USA).

### Ethical approval and data handling

The HAI program and the present study were conducted in accordance with the Declaration of Helsinki and approved by the Swedish Ethical Review Authority (Number 07-031 with extensions). All participants in the program provided written informed consent to participation in the health program and to subsequent research based on the collected outcome data. Individual informed consent was not obtained for the control population as they were identified through nationwide administrative registers and available to the researchers only as pseudonymized register data, as approved by the Swedish Ethical Review Authority. All data handling complied with applicable data protection regulations.

Generative artificial intelligence (ChatGPT, GPT-5; OpenAI, San Francisco, CA, USA) was used to assist with the development of statistical scripts in R and to improve clarity and language in the text. Following the use of this tool, the authors critically reviewed, edited and verified all content. The authors retain full responsibility for the study design, analyses, interpretation of the data, and the final manuscript.

## Results

### Study population

The exclusion process resulted in the exclusion of 626 (9.4%) HAI participants and 8,898 (13.4%) controls with prevalent diabetes at baseline. An additional 5 (<0.1%) HAI participants and 41 (<0.1%) controls were excluded due to missing educational data; see Fig 1. The final study population comprised 63,561 individuals, including 6,018 HAI participants and 57,543 matched controls. Mean follow-up was 4.9 years (standard deviation [SD] 2.9) in the HAI cohort and 4.8 years (SD 2.9) in the control cohort, with a total follow-up time of 29,714 and 276,761 person-years, respectively. During follow-up, incident diabetes occurred in 335 (5.6%) HAI participants and 3,919 (6.8%) controls. Baseline characteristics are presented in Table 1, with additional characteristics provided in S1 Table.

**Table 1:** Baseline characteristics of the study population after exclusion.

Variable	Total cohort (*N* = 63,561)	HAI^a^ cohort (*n* = 6,018)	Control cohort (*n* = 57,543)
Age, Mean (SD), years	70.4 (0.4)	70.4 (0.2)	70.4 (0.5)
Female sex, *n* (%)	34,418 (54.2)	3,237 (53.8)	31,181 (54.2)
Born in Sweden, *n* (%)	56,846 (89.4)	5,739 (95.4)	51,107 (88.8)
Home care services, *n* (%)	1,455 (2.3)	48 (0.8)	1,407 (2.5)
***Highest education, n (%)***			
<9 years of elementary school	4,136 (6.5)	404 (6.7)	3,732 (6.5)
9 years of elementary school	3,791 (6.0)	368 (6.1)	3,423 (6.0)
2 years of upper secondary school	17,560 (27.6)	1,684 (28.0)	15,876 (27.6)
>2 years of upper secondary school	38,074 (59.9)	3,562 (59.2)	34,512 (60.0)
***Diagnoses (ICD-10-codes*** ^***b***^ ***), n (%)***			
Myocardial infarction (I21)	2,050 (3.2)	213 (3.5)	1,837 (3.2)
Stroke (I61, I63, I64)	1,649 (2.6)	146 (2.4)	1,503 (2.6)
Angina pectoris (I20)	1,895 (3.0)	200 (3.3)	1,695 (3.0)
Renal disease (N17-N19)	523 (0.8)	29 (0.5)	494 (0.9)
Chronic obstructive pulmonary disease (J44)	1,547 (2.4)	80 (1.3)	1,467 (2.6)
Cancer of the colon or rectum (C18, C20)	716 (1.1)	69 (1.2)	647 (1.1)
Breast cancer (C50)	2,239 (3.5)	208 (3.5)	2,031 (3.5)
***Medication (ATC-code*** ^***c***^ ***), n (%)***			
Antihypertensives (C03, C07, C08, C09)	34,625 (54.5)	3,593 (59.7)	31,032 (53.9)
Lipid-lowering agents (C10)	19,276 (30.3)	2,195 (36.5)	17,081 (29.7)
Prednisolone (H02AB06)	7,704 (12.1)	495 (8.2)	7,209 (12.5)

Continuous variables are presented as mean (standard deviation [SD]) and categorical variables as number (%), where *n* is the number of participants with the respective characteristic and *N* is the number of participants in the respective cohort.

^a^HAI, Healthy Ageing Initiative.

^b^ICD-10, International Classification of Diseases, 10th Revision.

^c^ATC, Anatomical Therapeutic Chemical Classification System.

**Fig 1:**
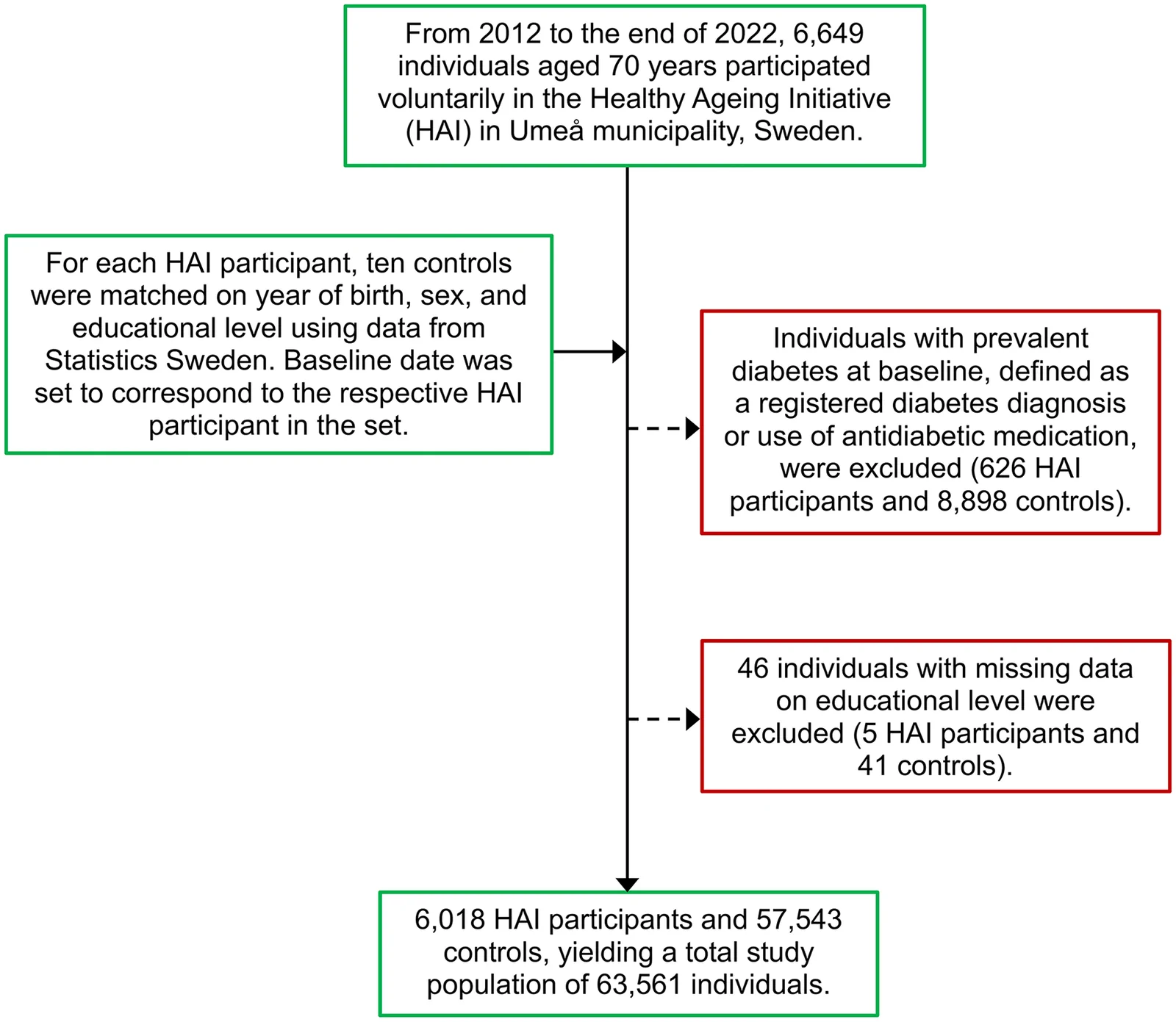
Flowchart of the study population selection process. HAI, Healthy Ageing Initiative.

### Primary analyses

Participation in the HAI program was associated with a lower risk of incident diabetes compared with controls (adjusted hazard ratio [HR] 0.76; 95% confidence interval [CI] [0.68,0.85]; *p* < 0.001), further detailed in the S4 Table. This association was robust in unadjusted (S2 and S3 Table) and adjusted sensitivity analyses (S5 Table), including a model accounting for the competing event of death (S6 Table). The association was consistent over time, with no indication of violation of the proportional hazard assumption (*p* = 0.78). Predicted absolute risk of diabetes, depicted in Fig 2, was 4.85% (95% CI [4.33,5.39]) in the HAI cohort at 5 years and 6.31% (95% CI [6.09,6.55]) among controls, corresponding to an absolute risk reduction of 1.46 percentage points (95% CI [0.91,1.98]; *p* < 0.001). At 10 years, the corresponding absolute risk difference was 3.42 percentage points (95% CI [2.13,4.75]; *p* < 0.001). These yielded numbers needed to treat of 68 (95% CI [50,110]) and 29 (95% CI [21,46]), respectively. When standardized to the 6,018 HAI participants, the model-predicted risks corresponded to approximately 380 expected cases based on the control risk and 292 predicted cases among HAI participants at 5 years, an estimated difference of 88 cases (95% CI [55,119]). At 10 years, the corresponding estimates were 938 and 732 cases, respectively, an estimated difference of 206 cases (95% CI [128,286]). Detailed cumulative risk analyses results are provided in S7 Table, and figures for observed risk in S1 Fig.

**Fig 2:**
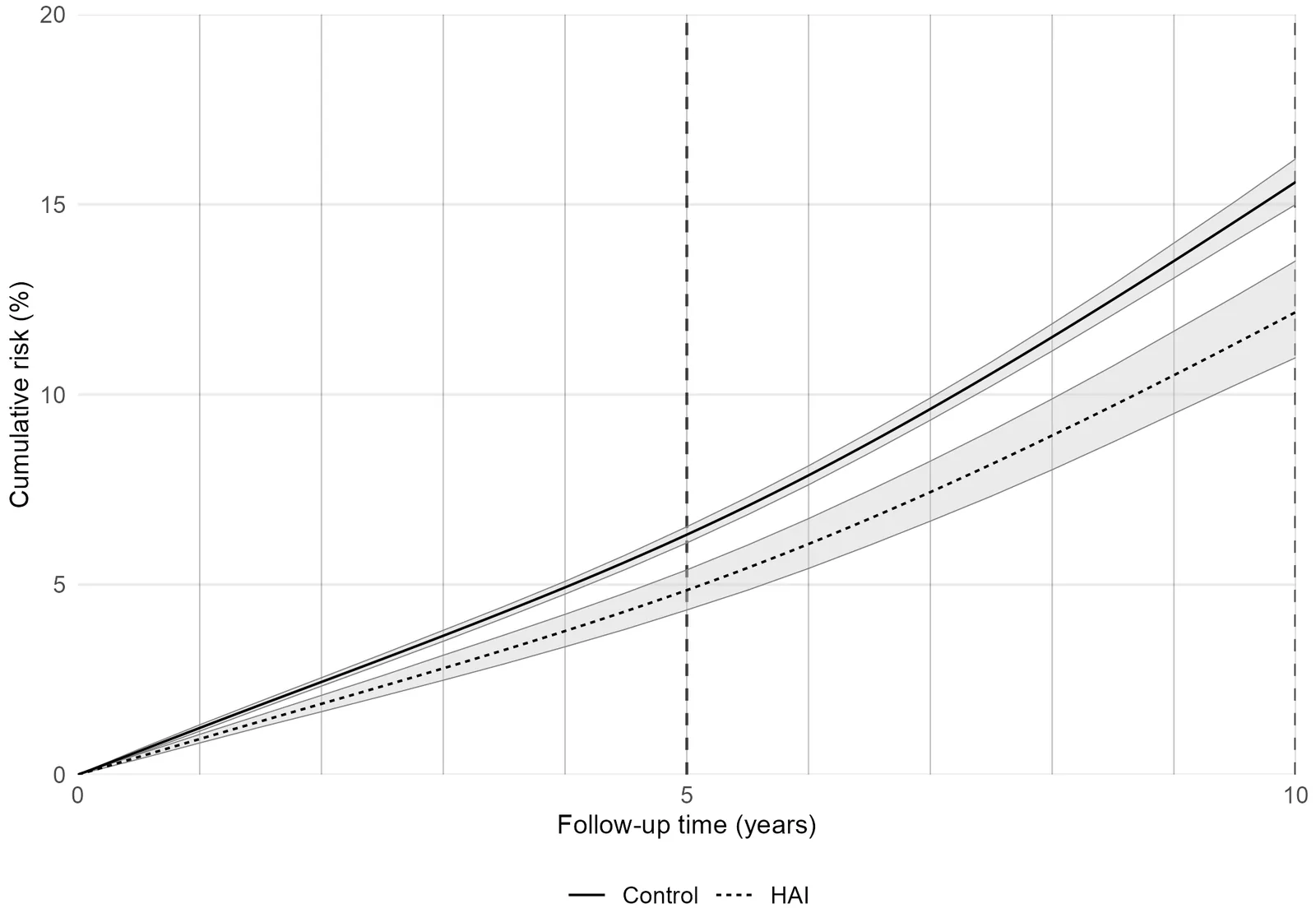
Predicted cumulative absolute risk of incident diabetes by study group. Risks were estimated using a multivariable flexible parametric survival model (Royston–Parmar). Shaded areas represent 95% confidence intervals derived from cluster bootstrap resampling of matched sets with model refitting. HAI, Healthy Ageing Initiative.

### Secondary analyses

The association between HAI participation and diabetes incidence was broadly consistent across examined subgroups. There was no statistically significant interaction by age, sex, country of birth, home care services, educational level, CVD, chronic obstructive pulmonary disease, or use of antihypertensive medication (Fig 3 and S8 Table). The only statistically significant interaction was observed for use of lipid-lowering agents (*p* for interaction = 0.008). The association was stronger among non-users (HR 0.65; 95% CI [0.55,0.77]) than among users (HR 0.88; 95% CI [0.76,1.02]), for whom the CI included the null. Population attributable fraction (PAF) estimates were similar across the cohorts and the total cohort. At 5 years, the estimate was 51.82% (95% CI [51.22,52.42]) in the HAI cohort, 49.05% (95% CI [48.87,49.22]) in the control cohort, and 49.09% (95% CI [48.92,49.26]) in the total cohort. Detailed PAF estimates are presented in the S9 Table.

**Fig 3:**
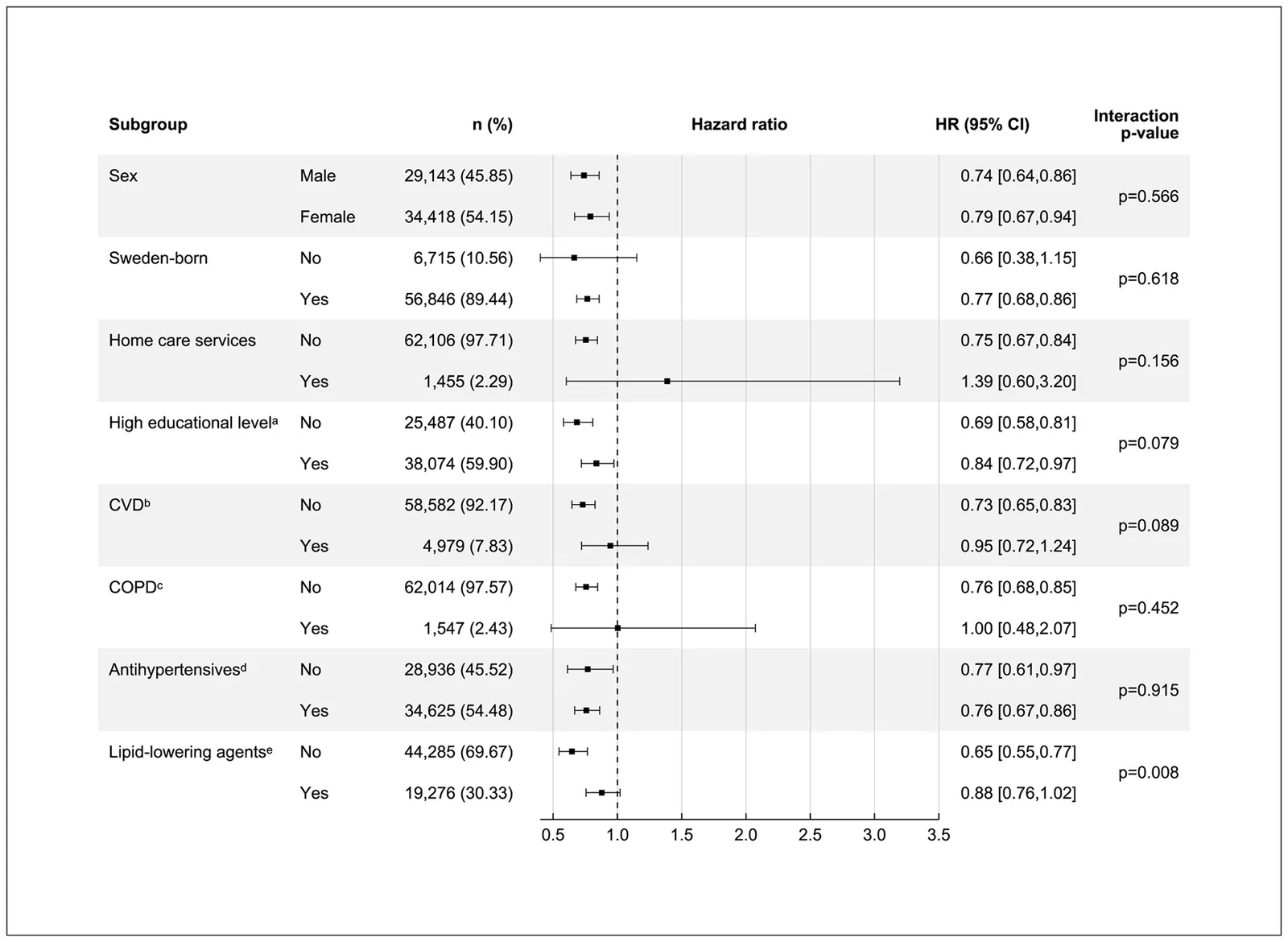
Subgroup-specific associations of participation in the Healthy Ageing Initiative with incident diabetes. Hazard ratios (HRs) and 95% confidence intervals (CIs) were estimated using separate adjusted Royston–Parmar models with robust standard errors clustered by matched set. HRs compare Healthy Ageing Initiative participants with controls within each subgroup level; values below 1 indicate a lower incidence of diabetes among participants. Squares represent HRs, horizontal lines represent 95% CIs. Interaction p-values were obtained using Wald tests. ICD-10,  International Statistical Classification of Diseases and Related Health Problems, 10th Revision. ATC,  Anatomical Therapeutic Chemical Classification System. ^a^High educational level was defined as >2 years of upper secondary school. ^b^CVD: cardiovascular disease, defined as ICD-10: I21, I61, I63, I64, and/or I20. ^c^COPD: Chronic obstructive pulmonary disease, ICD-10: J44. ^d^Antihypertensives defined as ATC-codes C03, C07, C08 and C09. ^e^Lipid-lowering agents defined as ATC-code C10.

## Discussion

In this population-based observational cohort study of older adults, participation in a low-intensity, population-wide preventive health assessment program was associated with a lower risk of incident diabetes stable over time, compared with matched controls. The association was consistent across multiple analytical approaches, including competing-risk analyses, and translated to a potentially clinically relevant absolute risk reduction and number needed to treat over 5 and 10 years. Further, the association with a lower incidence of diabetes was consistent across most examined subgroups.

While intensive lifestyle intervention programs such as the Diabetes Prevention Program and the Finnish Diabetes Prevention Study have demonstrated substantial reduction in diabetes incidence in high-risk individuals [10,12], their intensity and controlled delivery limit their direct translation to routine care. The present study suggests that lower-intensity, population-wide approaches may also be associated with lower diabetes incidence in routine-care settings. Other European and Nordic initiatives have demonstrated the favorable changes in lifestyle factors and cardiometabolic risk profiles, although primarily in more selected populations [16–18]. Our findings extend these observations by showing an association with incident diabetes in an older, population-based cohort. Unlike intensive high-risk interventions, the HAI did not rely on repeated follow-up or a protocol-driven lifestyle program, yet participation was associated with lower diabetes incidence. These findings suggest that relatively modest, individually tailored preventive efforts delivered at scale may have the potential to contribute to diabetes prevention at the population level.

The broadly consistent associations across the examined subgroups suggest that the program’s potential benefits may not be confined to specific demographic or clinical groups. This could support not restricting lifestyle interventions to groups already at a higher cardiometabolic risk. It is important to note that some of the estimates were imprecise, and the absence of a statistically significant interaction should not be directly interpreted as evidence of identical associations. Although a weaker association was found for individuals using lipid-lowering agents, these findings should be considered exploratory. Previous research on statins has demonstrated a small increased risk of diabetes and suggested less favorable changes in fasting glucose during lifestyle interventions [27,28]. However, use of lipid-lowering agents may also reflect a higher cardiometabolic burden at baseline, and better preventive efforts within primary care. Because multiple interaction tests were performed, these findings should be interpreted cautiously and not as definitive evidence that HAI was less beneficial among users of lipid-lowering agents.

PAF analyses indicated that a substantial proportion of incident diabetes cases was associated with a handful of selected baseline risk factors related to cardiometabolic risk. Although consistent in direction with previous population-based research [29,30], these results should be interpreted as exploratory as the model assumes causality, and baseline risk factors may act as markers for underlying disease burden rather than independent modifiable targets. Thus, the mechanisms underlying the observed associations are likely multifactorial. The HAI program could increase individual awareness of cardiometabolic risk and thereby further motivate lifestyle modification relevant for diabetes prevention. Such effects may be particularly relevant in older adults, where adherence to lifestyle interventions has been shown to be comparable to, or even greater than, that observed in younger populations [7,31].

There are important strengths of this study. The large population-based design, with participation from 54% of the eligible population, and a matched control population drawn from nationwide population registers, provides a broad comparison population and high statistical precision. This also increases the real-world aspect of the results and shows that the intervention is applicable in a semi-large scale. The use of nationwide registers for follow-up minimized loss to follow-up and ensured outcome ascertainment. Using multiple analytical approaches strengthened the robustness of the findings. But several limitations should also be acknowledged. The observational study design precludes causal inference and cannot exclude residual confounding. Participation in the HAI program was voluntary, raising the possibility of selection bias due to a “healthy-volunteer” effect [32]. The HAI cohort had more favorable values for some baseline characteristics, and the proportion excluded due to prevalent diabetes was lower than in the control group. Matching and multivariable adjusting cannot fully compensate for the potential effects of such bias. A total of 209 individuals initially included as controls subsequently participated in the HAI program and remained in the control cohort in the statistical analyses. This overlap could have introduced treatment contamination and attenuated the association towards the null if participation reduced their subsequent diabetes risk. The small number of individuals involved suggests that the potential downward bias may have been limited, although its magnitude was not quantified. Additionally, data on certain metabolic risk factors such as plasma glucose levels and body mass index, were not available for inclusion in the analyses and may have been relevant for the interpretation of the results. Diabetes diagnoses recorded exclusively in primary care were not available in the National Patient Register, and individuals managed without glucose-lowering medication may therefore have been identified later or not captured. Early events were not excluded in order to reflect real-world conditions.

Taken this into account, the findings suggest that preventive programs embedded in routine care and offered broadly to older adults may represent a feasible complement to intensive high-risk prevention strategies for diabetes. Given the alarming incidence of diabetes in aging populations, even modest risk reductions may translate into substantial absolute benefits at a population level. Future research should aim to evaluate the cost-effectiveness and implementation strategies of program with a similar overall design, as well as identify which components contribute most to the observed associations for more effective future program design. Future studies using other approaches could help clarify the intervention’s role and underlying mechanisms, to inform on optimization in prevention strategies for older adults.

In conclusion, participation in this population-wide, low-intensity preventive health assessment program aimed at lifestyle modification was associated with a lower risk of incident diabetes among older adults. However, more robust evidence is needed to determine the intervention’s contribution to the observed association, and to better account for potential healthy-volunteer effects. Nevertheless, these findings provide real-world support to the potential role of scalable, routine care-based prevention strategies in addressing the growing burden of diabetes in aging populations.

## Supporting information

S1 Appendix Description of the Healthy Ageing Initiative health program. (DOCX)

S1 Checklist Strengthening the reporting of observational studies in epidemiology (STROBE) Checklist. The STROBE checklist is best used in conjunction with this article (freely available on the Websites of PLoS Medicine at http://www.plosmedicine.org/, Annals of Internal Medicine at http://www.annals.org/, and Epidemiology at http://www.epidem.com/). Information on the STROBE Initiative is available at http://www.strobe-statement.org.
(DOCX)

S1 Fig Cumulative absolute risk of incident diabetes under alternate approaches to death. The shaded areas represent 95% confidence intervals. **A)** Estimated using the Kaplan–Meier method, with death treated as censoring. **B)** Estimated using the Fine-Gray competing risk method, with death treated as a competing event. HAI, Healthy Ageing Initiative.
(TIFF)

S1 Table Extended baseline characteristics of the study population after exclusion. (DOCX)

S2 Table Results from univariable Cox analyses of incident diabetes stratified by matched set. (DOCX)

S3 Table Results from univariable Cox and Fine-Gray analyses of incident diabetes without matched-set stratification. (DOCX)

S4 Table Results from the primary multivariable Royston–Parmar analysis of incident diabetes. (DOCX)

S5 Table Results from the Royston–Parmar sensitivity analysis of incident diabetes adjusted for cancer diagnoses. (DOCX)

S6 Table Results from unstratified multivariable Royston–Parmar and Fine-Gray sensitivity analyses of incident diabetes. (DOCX)

S7 Table Results from cumulative absolute risk analyses of incident diabetes at 5 and 10 years. (DOCX)

S8 Table Results from interaction analyses of Healthy Ageing Initiative participation and incident diabetes across baseline covariates. (DOCX)

S9 Table Results from population attributable fraction analyses of incident diabetes at 5 and 10 years. (DOCX)

## Abbreviations

CI: confidence interval
CVD: cardiovascular disease
DPS: Diabetes Prevention Study
HAI: Healthy Ageing Initiative
HR: hazard ratio
PAF: Population attributable fraction
SD: standard deviation
