# Supplementary material for: Association of a multiple risk factor assessment and intervention program with risk of diabetes: A population-based matched cohort study
Source: PLoS Med. 2026 Sep 21;23(9):e1005043. doi: 10.1371/journal.pmed.1005043 (PMC13626461; doi:10.1371/journal.pmed.1005043)
Supplement: Supplementary file 1 [file pmed.1005043.s001.docx]

**S1 Appendix.** Description of the Healthy Ageing Initiative health program.

**Program structure**

The Healthy Ageing Initiative (HAI) was delivered by research nurses at a clinic in two visits approximately one week apart. The program combined assessment of cardiometabolic health and lifestyle with individualized feedback and motivational counselling. The HAI program has been described previously [1]. The present appendix provides a concise description of the components relevant to this study.

**First visit: health and lifestyle assessment**

Participants attended the first visit at the clinic, receiving information about and consenting to participation in the program. They completed a questionnaire covering medical history, diet, physical activity, smoking, alcohol use, mood, cognitive function, and other health-related behaviours. The clinical assessment included height, weight, waist and hip circumference, seated blood pressure, fasting capillary blood glucose, and fasting blood lipids. Body composition was assessed using dual-energy X-ray absorptiometry (DXA), including measures of total and regional adiposity fat mass. Together with body mass index, waist circumference, metabolic measurements, and reported lifestyle, the results provided quantitative and visual support for discussions about adiposity, weight management, diet, and physical activity. The first visit lasted approximately three hours. Participants received initial feedback during the assessment and were subsequently provided with a hip-worn accelerometer to measure physical activity for seven days.

**Second visit: feedback and motivational counselling**

At the second visit, the same research nurse reviewed the combined assessment results with the participant, including blood pressure, glucose and lipid levels, anthropometric and DXA measurements, questionnaire responses, and daily physical activity patterns recorded by the accelerometer. These results formed the basis of an individualized motivational interview [2] focusing on relevant areas such as diet, weight management, physical activity, smoking, and alcohol use. Dietary counselling addressed the relationships between dietary habits and blood glucose, triglycerides, and cholesterol. Participants were encouraged to reduce foods rich in saturated fat and rapidly absorbed carbohydrates and to increase their intake of vegetables, fish, long-acting carbohydrates, and sources of polyunsaturated fat. Physical activity advice was adapted to the accelerometer results and the participant’s circumstances, with encouragement to meet recommendations for 150 minutes of moderate- or 75 minutes of vigorous-intensity weekly activity.

**Clinical follow-up**

Fasting capillary glucose values of ≥6.1 mmol/L at the first visit were reassessed at the second visit. Participants with glucose values of ≥7.0 mmol/L at both visits were advised to contact their general practitioner for further evaluation. Those with confirmed values of 6.1–6.9 mmol/L received lifestyle advice and were advised to arrange follow-up with their general practitioner after approximately three months. Clinically relevant elevations in blood pressure or lipid levels similarly resulted in recommendations for home monitoring, primary-care follow-up, or urgent assessment, depending on their severity and the participant’s comorbidities.

**References:**

1. Nordström A, Bergman J, Björk S, Carlberg B, Johansson J, Hult A, et al. A multiple risk factor program is associated with decreased risk of cardiovascular disease in 70-year-olds: A cohort study from Sweden. PLoS Med. 2020;17(6):e1003135. Epub 20200611. doi: 10.1371/journal.pmed.1003135. PubMed PMID: 32525878; PubMed Central PMCID: PMC7289341.
2. Miller WR, Rose GS. Toward a theory of motivational interviewing. Am Psychol. 2009 Sep;64(6):527–37. doi:10.1037/a0016830. PMID: 19739882; PMCID: PMC2759607.
