# Supplementary material for: Association of a multiple risk factor assessment and intervention program with risk of diabetes: A population-based matched cohort study
Source: PLoS Med. 2026 Sep 21;23(9):e1005043. doi: 10.1371/journal.pmed.1005043 (PMC13626461; doi:10.1371/journal.pmed.1005043)
Supplement: Supplementary file 2 [file pmed.1005043.s002.docx]

STROBE Statement—Checklist of items that should be included in reports of ***cohort studies***

|  | Item No | Recommendation | Page No |
| --- | --- | --- | --- |
| **Title and abstract** | 1 | (*a*) Indicate the study’s design with a commonly used term in the title or the abstract | (a) Title; Abstract |
|  |  | (*b*) Provide in the abstract an informative and balanced summary of what was done and what was found | (b) Abstract |
| Introduction | | | |
| Background/rationale | 2 | Explain the scientific background and rationale for the investigation being reported | Introduction, paragraphs 1-3 |
| Objectives | 3 | State specific objectives, including any prespecified hypotheses | Introduction, paragraph 4 |
| Methods | | | |
| Study design | 4 | Present key elements of study design early in the paper | Methods: Study design and setting |
| Setting | 5 | Describe the setting, locations, and relevant dates, including periods of recruitment, exposure, follow-up, and data collection | Methods: Study design and setting |
| Participants | 6 | (*a*) Give the eligibility criteria, and the sources and methods of selection of participants. Describe methods of follow-up | (a) Methods: Healthy Ageing Initiative cohort; Outcome definition |
|  |  | (*b*) For matched studies, give matching criteria and number of exposed and unexposed | (b) Methods: Control cohort. Results: Study population |
| Variables | 7 | Clearly define all outcomes, exposures, predictors, potential confounders, and effect modifiers. Give diagnostic criteria, if applicable | Methods: Outcome definition; Covariates |
| Data sources/ measurement | 8* | For each variable of interest, give sources of data and details of methods of assessment (measurement). Describe comparability of assessment methods if there is more than one group | Methods: Covariates |
| Bias | 9 | Describe any efforts to address potential sources of bias | Methods: Covariates |
| Study size | 10 | Explain how the study size was arrived at | Methods: Healthy Ageing Initiative cohort; Control cohort |
| Quantitative variables | 11 | Explain how quantitative variables were handled in the analyses. If applicable, describe which groupings were chosen and why | Not applicable |
| Statistical methods | 12 | (*a*) Describe all statistical methods, including those used to control for confounding | a) Methods: Statistical analysis, paragraphs1-4 |
|  |  | (*b*) Describe any methods used to examine subgroups and interactions | b) Methods: Statistical analysis, paragraph 3 |
|  |  | (*c*) Explain how missing data were addressed | c) Methods: Exclusion criteria |
|  |  | (*d*) If applicable, explain how loss to follow-up was addressed | d) Not applicable |
|  |  | (*e*) Describe any sensitivity analyses | e) Methods: Statistical analysis, paragraph 2 |
| Results | | |  |
| Participants | 13* | (a) Report numbers of individuals at each stage of study—eg numbers potentially eligible, examined for eligibility, confirmed eligible, included in the study, completing follow-up, and analysed | a) Results: Study population |
|  |  | (b) Give reasons for non-participation at each stage | b) Not applicable |
|  |  | (c) Consider use of a flow diagram | c) Results: Fig 1 |
| Descriptive data | 14* | (a) Give characteristics of study participants (eg demographic, clinical, social) and information on exposures and potential confounders | a) Results: Table 1 Supporting Information: S1 Table |
|  |  | (b) Indicate number of participants with missing data for each variable of interest | b) Results: Study population |
|  |  | (c) Summarise follow-up time (eg, average and total amount) | c) Results: Study population |
| Outcome data | 15* | Report numbers of outcome events or summary measures over time | Results: Study population |

| Main results | 16 | (*a*) Give unadjusted estimates and, if applicable, confounder-adjusted estimates and their precision (eg, 95% confidence interval). Make clear which confounders were adjusted for and why they were included | a) Results: Primary analyses Methods: Covariates |
| --- | --- | --- | --- |
|  |  | (*b*) Report category boundaries when continuous variables were categorized | b) Not applicable |
|  |  | (*c*) If relevant, consider translating estimates of relative risk into absolute risk for a meaningful time period | c) Results: Primary analyses; Fig 2 |
| Other analyses | 17 | Report other analyses done—eg analyses of subgroups and interactions, and sensitivity analyses | Results: Secondary analyses; Fig 3 |
| Discussion | | | |
| Key results | 18 | Summarise key results with reference to study objectives | Discussion: paragraphs 1, 2, 6, 7 |
| Limitations | 19 | Discuss limitations of the study, taking into account sources of potential bias or imprecision. Discuss both direction and magnitude of any potential bias | Discussion: paragraph 5 |
| Interpretation | 20 | Give a cautious overall interpretation of results considering objectives, limitations, multiplicity of analyses, results from similar studies, and other relevant evidence | Discussion: paragraph 6, 7 |
| Generalisability | 21 | Discuss the generalisability (external validity) of the study results | Discussion: paragraphs 2, 3, 5. |
| Other information | | | |
| Funding | 22 | Give the source of funding and the role of the funders for the present study and, if applicable, for the original study on which the present article is based | Financial disclosure |

*Give information separately for exposed and unexposed groups.

**Note:** An Explanation and Elaboration article discusses each checklist item and gives methodological background and published examples of transparent reporting. The STROBE checklist is best used in conjunction with this article (freely available on the Web sites of PLoS Medicine at http://www.plosmedicine.org/, Annals of Internal Medicine at http://www.annals.org/, and Epidemiology at http://www.epidem.com/). Information on the STROBE Initiative is available at http://www.strobe-statement.org.
