# Supplementary material for: Association of a multiple risk factor assessment and intervention program with risk of diabetes: A population-based matched cohort study
Source: PLoS Med. 2026 Sep 21;23(9):e1005043. doi: 10.1371/journal.pmed.1005043 (PMC13626461; doi:10.1371/journal.pmed.1005043)
Supplement: Supplementary file 3 [file pmed.1005043.s004.docx]

**S1 Table. Extended baseline characteristics of the study population after exclusion.**

Continuous variables are presented as mean (standard deviation [SD]) and categorical variables as n (%), where n is the number of participants in the respective category and N is the number of participants in the respective cohort. P-values for between-group comparisons were calculated using Welch´s t-test for continuous variables and Pearson´s X^2^ test for categorical variables. For educational level, a global p-value was estimated using Pearson´s X^2^ test.

| **Variable** | **Total cohort (N = 63,561)** | **HAI^a^ cohort**  **(n = 6,018)** | **Control cohort**  **(n = 57,543)** | **P-value** |
| --- | --- | --- | --- | --- |
| Age, Mean (SD), years | 70.4 (0.4) | 70.4 (0.2) | 70.4 (0.5) | 0.195 |
| Female sex, n (%) | 34,418 (54.2) | 3,237 (53.8) | 31,181 (54.2) | 0.555 |
| Born in Sweden, n (%) | 56,846 (89.4) | 5,739 (95.4) | 51,107 (88.8) | <0.001 |
| Home care services, n (%) | 1,455 (2.3) | 48 (0.8) | 1,407 (2.5) | <0.001 |
| ***Highest education, n (%)*** |  |  |  |  |
| <9 years of elementary school | 4,136 (6.5) | 404 (6.7) | 3,732 (6.5) |  |
| 9 years of elementary school | 3,791 (6.0) | 368 (6.1) | 3,423 (6.0) |  |
| 2 years of upper secondary school | 17,560 (27.6) | 1,684 (28.0) | 15,876 (27.6) |  |
| > 2 years of upper secondary school | 38,074 (59.9) | 3,562 (59.2) | 34,512 (60.0) |  |
| Global P-value |  |  |  | 0.670 |
| ***Diagnoses (ICD-10-codes^b^), n (%)*** |  |  |  |  |
| Myocardial infarction (I21) | 2,050 (3.2) | 213 (3.5) | 1,837 (3.2) | 0.147 |
| Stroke (I61, I63, I64) | 1,649 (2.6) | 146 (2.4) | 1,503 (2.6) | 0.388 |
| Angina pectoris (I20) | 1,895 (3.0) | 200 (3.3) | 1,695 (3.0) | 0.101 |
| Renal disease (N17-N19) | 523 (0.8) | 29 (0.5) | 494 (0.9) | 0.002 |
| Chronic obstructive pulmonary disease (J44) | 1,547 (2.4) | 80 (1.3) | 1,467 (2.6) | <0.001 |
| Cancer of the colon or rectum (C18, C20) | 716 (1.1) | 69 (1.2) | 647 (1.1) | 0.877 |
| Breast cancer (C50) | 2,239 (3.5) | 208 (3.5) | 2,031 (3.5) | 0.769 |
| Fracture (S12-S82, excluding S62) | 10,031 (15.8) | 1,019 (16.9) | 9,012 (15.7) | 0.011 |
| Parkinson’s disease (G20) | 348 (0.6) | 35 (0.6) | 313 (0.5) | 0.706 |
| Mental and behavioural disorders due to use of alcohol (F10) | 1,183 (1.9) | 49 (0.8) | 1,134 (2.0) | <0.001 |
| ***Medication (ATC-code^c^), n (%)*** |  |  |  |  |
| Antihypertensives (C03, C07, C08, C09) | 34,625 (54.5) | 3,593 (59.7) | 31,032 (53.9) | <0.001 |
| Lipid-lowering agents (C10) | 19,276 (30.3) | 2,195 (36.5) | 17,081 (29.7) | <0.001 |
| Anticoagulants (B01) | 20,460 (32.2) | 1,934 (32.1) | 18,526 (32.2) | 0.927 |
| Antidepressants (N06A) | 11,804 (18.6) | 1,087 (18.1) | 10,717 (18.6) | 0.286 |
| Sedatives (N05C) | 15,080 (23.7) | 1,366 (22.7) | 13,714 (23.8) | 0.049 |
| Prednisolone (H02AB06) | 7,704 (12.1) | 495 (8.2) | 7,209 (12.5) | <0.001 |
| Levothyroxine (H03AA01) | 5,609 (8.8) | 495 (8.2) | 5,114 (8.9) | 0.085 |

*^a^ HAI = Healthy Ageing Initiative*

*^b^ ICD-10 = International Classification of Diseases, 10^th^ Revision
^c^ ATC = Anatomical Therapeutic Chemical Classification System*
