# Supplementary material for: Association of a multiple risk factor assessment and intervention program with risk of diabetes: A population-based matched cohort study
Source: PLoS Med. 2026 Sep 21;23(9):e1005043. doi: 10.1371/journal.pmed.1005043 (PMC13626461; doi:10.1371/journal.pmed.1005043)
Supplement: Supplementary file 4 [file pmed.1005043.s005.docx]

**S2 Table. Results from univariable Cox analyses of incident diabetes stratified by matched set.**

Hazard ratios (HRs) with 95% confidence intervals (CIs) were estimated using univariable Cox proportional hazards regression models. P-values were derived from Wald tests. The proportional hazards (PH) assumption was assessed using Schoenfeld residuals, with the corresponding p-values reported.

| **Variable** | **HR (95% CI)** | **P-value** | **PH p-value** |
| --- | --- | --- | --- |
| **Main effect of intervention** | 0.80 [0.71,0.90] | <0.001 | 0.781 |
| Age (per year) | 0.96 [0.85,1.07] | 0.452 | 0.267 |
| Female sex | Matched on |  | Matched on |
| Born in Sweden | 0.79 [0.71,0.87] | <0.001 | 0.464 |
| Home care services | 1.44 [1.17,1.78] | <0.001 | 0.707 |
| ***Highest education*** | Matched on |  | Matched on |
| ***Diagnoses (ICD-10-codes^a^)*** |  |  |  |
| Myocardial infarction (I21) | 1.99 [1.74,2.29] | <0.001 | 0.049 |
| Stroke (I61, I63, I64) | 1.33 [1.11,1.59] | 0.002 | 0.391 |
| Angina pectoris (I20) | 1.70 [1.47,1.97] | <0.001 | 1.000 |
| Renal disease (N17-N19) | 1.56 [1.16,2.09] | 0.003 | 0.045 |
| Chronic obstructive pulmonary disease (J44) | 1.57 [1.30,1.88] | <0.001 | 0.402 |
| Cancer of the colon or rectum (C18, C20) | 1.09 [0.80,1.49] | 0.569 | 0.538 |
| Breast cancer (C50) | 1.49 [1.25,1.78] | <0.001 | 0.558 |
| Fracture (S12-S82, excluding S62) | 0.95 [0.86,1.04] | 0.260 | 0.590 |
| Mental and behavioural disorders due to use of alcohol (F10) | 1.17 [0.94,1.47] | 0.164 | 0.299 |
| Parkinson’s disease (G20) | 0.67 [0.38,1.19] | 0.173 | 0.917 |
| ***Medication (ATC-code^b^)*** |  |  |  |
| Antihypertensives (C03, C07, C08, C09) | 2.25 [2.10,2.42] | <0.001 | 0.049 |
| Lipid lowering agents (C10) | 1.79 [1.68,1.91] | <0.001 | 0.002 |
| Anticoagulants (B01) | 1.71 [1.61,1.83] | <0.001 | 0.009 |
| Antidepressants (N06A) | 1.33 [1.22,1.44] | <0.001 | 0.261 |
| Sedatives (N05C) | 1.22 [1.13,1.31] | <0.001 | 0.167 |
| Prednisolone (H02AB06) | 1.46 [1.33,1.61] | <0.001 | 0.695 |
| Levothyroxine (H03AA01) | 1.24 [1.11,1.39] | <0.001 | 0.266 |

*^a^ ICD-10 = International Classification of Diseases, 10^th^ Revision*

*^b^ ATC = Anatomical Therapeutic Chemical Classification System*
