# Supplementary material for: Association of a multiple risk factor assessment and intervention program with risk of diabetes: A population-based matched cohort study
Source: PLoS Med. 2026 Sep 21;23(9):e1005043. doi: 10.1371/journal.pmed.1005043 (PMC13626461; doi:10.1371/journal.pmed.1005043)
Supplement: Supplementary file 5 [file pmed.1005043.s006.docx]

**S3 Table. Results from univariable Cox and Fine-Gray analyses of incident diabetes without matched-set stratification.**

Hazard ratios (HRs) from Cox proportional hazards regression models and subdistribution hazard ratios (sHRs) from Fine-Gray (FG) subdistribution hazard regression models are presented with 95% confidence intervals (CIs). In the Fine-Gray models, death was treated as a competing event. P-values are derived from Wald tests. Global p-value for education was estimated with a likelihood ratio test. The proportional hazards (PH) assumption was assessed for the Cox models using Schoenfeld residuals with the corresponding p-values reported.

| **Variable** | **HR (95% CI)** | **Cox p-value** | **sHR (95%CI)** | **FG p-value** | **PH p-value** |
| --- | --- | --- | --- | --- | --- |
| **Main effect of intervention** | 0.79 [0.71,0.89] | <0.001 | 0.82 [0.73,0.91] | <0.001 | 0.740 |
| Age (per year) | 0.93 [0.86,1.00] | 0.041 | 0.93 [0.86,1.00] | 0.057 | 0.201 |
| Female sex | 0.66 [0.62,0.70] | <0.001 | 0.67 [0.63,0.71] | <0.001 | 0.268 |
| Born in Sweden | 0.81 [0.74,0.89] | <0.001 | 0.81 [0.74,0.89] | <0.001 | 0.411 |
| Home care services | 1.48 [1.22,1.80] | <0.001 | 1.08 [0.89,1.31] | 0.455 | 0.715 |
| ***Highest education*** |  |  |  |  |  |
| < 9 years of elementary school | ref | ref | ref | ref | ref |
| 9 years of elementary school | 0.97 [0.83,1.12] | 0.639 | 0.95 [0.82,1.11] | 0.527 | 0.050 |
| 2 years of upper secondary school | 0.87 [0.78,0.97] | 0.009 | 0.87 [0.79,0.97] | 0.011 | 0.050 |
| >2 years of upper secondary school | 0.72 [0.65,0.79] | <0.001 | 0.73 [0.66,0.81] | <0.001 | 0.050 |
| Global P-value |  | <0.001 |  | <0.001 |  |
| ***Diagnoses (ICD-10-codes^a^)*** |  |  |  |  |  |
| Myocardial infarction (I21) | 2.26 [2.01,2.55] | <0.001 | 2.18 [1.94,2.46] | <0.001 | 0.536 |
| Stroke (I61, I63, I64) | 1.45 [1.23,1.71] | <0.001 | 1.33 [1.13,1.57] | <0.001 | 0.433 |
| Angina pectoris (I20) | 1.85 [1.62,2.11] | <0.001 | 1.80 [1.58,2.06] | <0.001 | 0.868 |
| Renal disease (N17-N19) | 1.91 [1.47,2.49] | <0.001 | 1.65 [1.26,2.17] | <0.001 | 0.021 |
| Chronic obstructive pulmonary disease (J44) | 1.61 [1.36,1.90] | <0.001 | 1.37 [1.16,1.62] | <0.001 | 0.716 |
| Cancer of the colon or rectum (C18, C20) | 1.12 [0.84,1.50] | 0.444 | 1.02 [0.76,1.36] | 0.913 | 0.906 |
| Breast cancer (C50) | 1.14 [0.97,1.34] | 0.103 | 1.11 [0.95,1.31] | 0.184 | 0.738 |
| Fracture (S12-S82, excluding S62) | 0.89 [0.81,0.97] | 0.009 | 0.86 [0.79,0.94] | 0.001 | 0.897 |
| Mental and behavioural disorders due to use of alcohol (F10) | 1.44 [1.17,1.78] | <0.001 | 1.24 [1.00,1.53] | 0.050 | 0.394 |
| Parkinson’s disease (G20) | 0.69 [0.40,1.18] | 0.176 | 0.57 [0.33,0.99] | 0.046 | 0.935 |
| ***Medication (ATC-code^b^)*** |  |  |  |  |  |
| Antihypertensives (C03, C07, C08, C09) | 2.33 [2.18,2.49] | <0.001 | 2.27 [2.13,2.43] | <0.001 | 0.024 |
| Lipid lowering agents (C10) | 1.90 [1.79,2.02] | <0.001 | 1.87 [1.76,1.99] | <0.001 | 0.004 |
| Anticoagulants (B01) | 1.82 [1.72,1.94] | <0.001 | 1.76 [1.65,1.87] | <0.001 | 0.004 |
| Antidepressants (N06A) | 1.22 [1.13,1.32] | <0.001 | 1.17 [1.08,1.27] | <0.001 | 0.240 |
| Sedatives (N05C) | 1.13 [1.06,1.22] | <0.001 | 1.10 [1.03,1.18] | 0.008 | 0.245 |
| Prednisolone (H02AB06) | 1.43 [1.31,1.56] | <0.001 | 1.36 [1.25,1.49] | <0.001 | 0.761 |
| Levothyroxine (H03AA01) | 1.09 [0.98,1.21] | 0.110 | 1.09 [0.98,1.21] | 0.095 | 0.513 |

*^a^ ICD-10 = International Classification of Diseases, 10^th^ Revision*

*^b^ ATC = Anatomical Therapeutic Chemical Classification System*
