# Supplementary material for: Association of a multiple risk factor assessment and intervention program with risk of diabetes: A population-based matched cohort study
Source: PLoS Med. 2026 Sep 21;23(9):e1005043. doi: 10.1371/journal.pmed.1005043 (PMC13626461; doi:10.1371/journal.pmed.1005043)
Supplement: Supplementary file 7 [file pmed.1005043.s008.docx]

**S5 Table.** **Results from the Royston-Parmar sensitivity analysis of incident diabetes adjusted for cancer diagnoses.**

Hazard ratios (HRs) with 95% confidence intervals (CIs) were estimated using a multivariable Royston-Parmar flexible parametric survival model with 4 degrees of freedom for baseline log cumulative hazard, and with robust standard errors clustered by matched set. The model included additional adjustment for cancer diagnoses. P-values are derived from Wald tests, and a global P-value for education is estimated using a likelihood ratio test.

| **Covariate** | **HR (95% CI)** | **P-value** |
| --- | --- | --- |
| **Main effect of intervention** | 0.76 [0.68,0.85] | <0.001 |
| Age (per year) | 0.92 [0.85,0.99] | 0.022 |
| Female sex | 0.66 [0.62,0.70] | <0.001 |
| Born in Sweden | 0.83 [0.76,0.91] | <0.001 |
| Home care services | 1.15 [0.94,1.40] | 0.176 |
| ***Highest education*** |  |  |
| < 9 years of elementary school | ref | ref |
| 9 years of elementary school | 0.99 [0.85,1.15] | 0.879 |
| 2 years of upper secondary school | 0.98 [0.88,1.09] | 0.679 |
| >2 years of upper secondary school | 0.80 [0.73,0.89] | <0.001 |
| Global P-value for education |  | <0.001 |
| ***Diagnoses (ICD-10-codes^a^)*** |  |  |
| Myocardial infarction (I21) | 1.25 [1.10,1.42] | <0.001 |
| Stroke (I61, I63, I64) | 0.92 [0.78,1.09] | 0.345 |
| Angina pectoris (I20) | 1.01 [0.88,1.16] | 0.873 |
| Renal disease (N17- N19) | 1.17 [0.90,1.53] | 0.244 |
| Chronic obstructive pulmonary disease (J44) | 1.19 [1.01,1.42] | 0.043 |
| Cancer of the colon or rectum (C18, C20) | 1.08 [0.81,1.44] | 0.615 |
| Breast cancer (C50) | 1.44 [1.22,1.69] | <0.001 |
| Mental and behavioural disorders due to use of alcohol (F10) | 1.07 [0.86,1.33] | 0.542 |
| ***Medication (ATC-code^b^)*** |  |  |
| Antihypertensives (C03, C07, C08, C09) | 1.94 [1.80,2.08] | <0.001 |
| Lipid lowering agents (C10) | 1.38 [1.28,1.47] | <0.001 |
| Antidepressants (N06A) | 1.15 [1.06,1.24] | <0.001 |
| Prednisolone (H02AB06) | 1.26 [1.15,1.38] | <0.001 |

*^a^ ICD-10 = International Classification of Diseases, 10^th^ Revision*

*^b^ ATC = Anatomical Therapeutic Chemical Classification System*
