# Supplementary material for: Association of a multiple risk factor assessment and intervention program with risk of diabetes: A population-based matched cohort study
Source: PLoS Med. 2026 Sep 21;23(9):e1005043. doi: 10.1371/journal.pmed.1005043 (PMC13626461; doi:10.1371/journal.pmed.1005043)
Supplement: Supplementary file 8 [file pmed.1005043.s009.docx]

**S6 Table. Results from unstratified multivariable Royston-Parmar and Fine-Gray sensitivity analyses of incident diabetes.**

Hazard ratios (HRs) from a Royston-Parmar (RP) flexible parametric survival model and subdistribution hazard ratios (sHRs) from a Fine-Gray (FG) subdistribution hazard model are presented with 95% confidence intervals (CIs). Both models were fitted without stratification. Death was treated as a competing event in the FG model. The RP model uses 4 degrees of freedom for baseline log cumulative hazard. P-values are derived from Wald tests, and a global P-value for educational level was estimated using a likelihood ratio test.

| **Covariate** | **HR (95% CI)** | **RP p-value** | **sHR (95%CI)** | **FG p-value** |
| --- | --- | --- | --- | --- |
| **Main effect of intervention** | 0.76 [0.68,0.85] | <0.001 | 0.78 [0.70,0.87] | <0.001 |
| Age (per year) | 0.92 [0.86,0.99] | 0.023 | 0.92 [0.86,0.99] | 0.031 |
| Female sex | 0.68 [0.63,0.72] | <0.001 | 0.69 [0.65,0.74] | <0.001 |
| Born in Sweden | 0.83 [0.76,0.91] | <0.001 | 0.83 [0.76,0.91] | <0.001 |
| Home care services | 1.15 [0.94,1.40] | 0.179 | 0.86 [0.71,1.06] | 0.154 |
| ***Highest education*** |  |  |  |  |
| < 9 years of elementary school | ref | ref | ref | ref |
| 9 years of elementary school | 0.99 [0.86,1.15] | 0.909 | 0.98 [0.84,1.13] | 0.746 |
| 2 years of upper secondary school | 0.98 [0.88,1.09] | 0.705 | 0.97 [0.88,1.08] | 0.636 |
| >2 years of upper secondary school | 0.81 [0.73,0.89] | <0.001 | 0.81 [0.74,0.90] | <0.001 |
| Global P-value education |  | <0.001 |  | <0.001 |
| ***Diagnoses (ICD-10-codes^a^)*** |  |  |  |  |
| Myocardial infarction (I21) | 1.25 [1.10,1.42] | <0.001 | 1.24 [1.09,1.41] | 0.001 |
| Stroke (I61, I63, I64) | 0.92 [0.78,1.09] | 0.344 | 0.88 [0.75,1.05] | 0.158 |
| Angina pectoris (I20) | 1.01 [0.88,1.16] | 0.852 | 1.01 [0.88,1.16] | 0.918 |
| Renal disease (N17- N19) | 1.17 [0.90,1.53] | 0.245 | 1.06 [0.81,1.39] | 0.680 |
| Chronic obstructive pulmonary disease (J44) | 1.20 [1.01,1.43] | 0.037 | 1.06 [0.89,1.26] | 0.537 |
| Mental and behavioural disorders due to use of alcohol (F10) | 1.07 [0.86,1.33] | 0.540 | 0.97 [0.78,1.21] | 0.804 |
| ***Medication (ATC-code^b^)*** |  |  |  |  |
| Antihypertensives (C03, C07, C08, C09) | 1.94 [1.80,2.09] | <0.001 | 1.91 [1.78,2.06] | <0.001 |
| Lipid lowering agents (C10) | 1.37 [1.28,1.47] | <0.001 | 1.38 [1.29,1.48] | <0.001 |
| Antidepressants (N06A) | 1.15 [1.06,1.25] | <0.001 | 1.12 [1.04,1.21] | 0.005 |
| Prednisolone (H02AB06) | 1.26 [1.15,1.38] | <0.001 | 1.22 [1.11,1.34] | <0.001 |

*^a^ ICD-10 = International Classification of Diseases, 10^th^ Revision*

*^b^ ATC = Anatomical Therapeutic Chemical Classification System*
