# Supplementary material for: Association of a multiple risk factor assessment and intervention program with risk of diabetes: A population-based matched cohort study
Source: PLoS Med. 2026 Sep 21;23(9):e1005043. doi: 10.1371/journal.pmed.1005043 (PMC13626461; doi:10.1371/journal.pmed.1005043)
Supplement: Supplementary file 9 [file pmed.1005043.s010.docx]

**S7 Table. Results from cumulative absolute risk analyses of incident diabetes at 5 and 10 years.**

Observed cumulative risks at 5 and 10 years were estimated using the Kaplan-Meier (KM) and Fine-Gray (FG) methods, with the latter treating death as a competing event. Predicted cumulative risk was estimated using a Royston-Parmar (RP) model accounting for clustering by matched set. Results are presented as cumulative risk for each group, risk difference (control minus HAI), p-value for risk difference, 95% confidence intervals (CIs), and number needed to treat (NNT). Confidence intervals for cumulative risk were obtained from the respective models, whereas confidence intervals and p-values for risk differences were obtained using bootstrap resampling. The log-rank test for the KM analysis and Gray´s test for the FG analysis both indicated an overall difference between the groups across the entire follow-up period (both *p*<0.001).

| **Method** | **Time (years)** | **HAI^a^ risk, % (95% CI)** | **Control risk, % (95% CI)** | **Risk difference, % (95% CI)** | **P-value** | **NNT (95% CI)** |
| --- | --- | --- | --- | --- | --- | --- |
| **KM** | **5** | 4.94 [4.30,5.58] | 6.25 [6.01,6.48] | 1.31 [0.65,1.99] | <0.001 | 76 [50,154] |
|  | **10** | 12.09 [10.25,13.90] | 15.41 [14.69,16.12] | 3.32 [1.23,5.29] | 0.004 | 30 [18,81] |
| **FG** | **5** | 4.88 [4.24,5.51] | 6.05 [5.82,6.28] | 1.18 [0.51,1.85] | <0.001 | 85 [53,195] |
|  | **10** | 11.53 [9.85,13.22] | 14.12 [13.50,14.74] | 2.59 [0.68,4.36] | 0.006 | 38 [22,146] |
| **RP** | **5** | 4.85 [4.33,5.39] | 6.31 [6.09,6.55] | 1.46 [0.91,1.98] | <0.001 | 68 [50,110] |
|  | **10** | 12.17 [10.97,13.52] | 15.59 [15.00,16.20] | 3.42 [2.13,4.75] | <0.001 | 29 [21,46] |

*^a^ HAI = Healthy Ageing Initiative.*
