# Supplementary material for: Association of a multiple risk factor assessment and intervention program with risk of diabetes: A population-based matched cohort study
Source: PLoS Med. 2026 Sep 21;23(9):e1005043. doi: 10.1371/journal.pmed.1005043 (PMC13626461; doi:10.1371/journal.pmed.1005043)
Supplement: Supplementary file 10 [file pmed.1005043.s011.docx]

**S8 Table. Results from interaction analyses of Healthy Ageing Initiative participation and incident diabetes across baseline covariates.**

Estimates are derived from an adjusted Royston-Parmar model clustered on matched set. Hazard ratios (HRs) with 95% confidence intervals (CIs) represent the estimated effect of participation in the Healthy Ageing Initiative program compared to controls within each subgroup defined by baseline covariates. Educational level is presented as binary and in four categories. P-values for multiplicative interactions are based on Wald tests, except for the global p-value for education which is based on a likelihood ratio test.

| **Covariate** | **Unexposed HR (95% CI)** | **Exposed HR (95% CI)** | **Interaction p-value** |
| --- | --- | --- | --- |
| Age (per year) | 0.76 [0.68,0.85] | 0.70 [0.41,1.21] | 0.757 |
| Female sex | 0.74 [0.64,0.86] | 0.79 [0.67,0.94] | 0.566 |
| Born in Sweden | 0.66 [0.38,1.15] | 0.77 [0.68,0.86] | 0.618 |
| Home care services | 0.75 [0.67,0.84] | 1.39 [0.60,3.20] | 0.156 |
| ***Educational level (binary)*** |  |  |  |
| High educational level^a^ | 0.69 [0.58,0.81] | 0.84 [0.72,0.97] | 0.079 |
| ***Educational level (per category)*** |  |  |  |
| < 9 years of elementary school (ref) | 0.64 [0.45,0.90] |  |  |
| 9 years of elementary school | 1.03 [0.71,1.52] | 1.63 [0.97,2.73] | 0.064 |
| 2 years of upper secondary school | 0.63 [0.51,0.79] | 1.00 [0.66,1.50] | 0.987 |
| >2 years of upper secondary school | 0.84 [0.72,0.97] | 1.32 [0.90,1.92] | 0.153 |
| Global p-value education |  |  | 0.052 |
| ***Diagnoses (ICD-10-codes^b^)*** |  |  |  |
| CVD^c^ | 0.73 [0.65,0.83] | 0.95 [0.72,1.24] | 0.089 |
| Chronic obstructive pulmonary disease (J44) | 0.76 [0.68,0.85] | 1.00 [0.48,2.07] | 0.452 |
| ***Medication (ATC-code^d^)*** |  |  |  |
| Antihypertensives (C03, C07–C09) | 0.77 [0.61,0.97] | 0.76 [0.67,0.86] | 0.915 |
| Lipid lowering agents (C10) | 0.65 [0.55,0.77] | 0.88 [0.76,1.02] | 0.008 |

*^a^ High educational level defined as >2 years of upper secondary school as binary compared to the combined other educational levels.*

*^b^ ICD-10 = International Classification of Diseases, 10^th^ Revision*

*^c^ CVD = cardiovascular disease marker constructed through combining the existence of one or more of Myocardial infarction (ICD-10 I21), Stroke (ICD-10 I61, I63, I64), and Angina pectoris (ICD-10 I20).*

*^d^ ATC = Anatomical Therapeutic Chemical Classification System*
