# Supplementary material for: Association of a multiple risk factor assessment and intervention program with risk of diabetes: A population-based matched cohort study
Source: PLoS Med. 2026 Sep 21;23(9):e1005043. doi: 10.1371/journal.pmed.1005043 (PMC13626461; doi:10.1371/journal.pmed.1005043)
Supplement: Supplementary file 11 [file pmed.1005043.s012.docx]

**S9 Table. Results from population attributable fraction analyses of incident diabetes at 5 and 10 years.**

Population attributable fractions (PAFs) for five selected baseline exposures were estimated individually and jointly using an adjusted Royston-Parmar model clustered on matched set. 95% confidence intervals (CIs) are provided. The PAF represents the proportion of incident diabetes that theoretically could have been prevented if the respective exposure had been eliminated, assuming a causal relationship. Absolute risk reduction (ARR) is calculated as the absolute difference between the adjusted observed cumulative risk and the counterfactual risk under elimination of the exposure.

**Panel A. Healthy Ageing Initiative cohort**

The adjusted observed cumulative risk was 5.13% at 5 years and 12.32% at 10 years. Under the joint counterfactual elimination of all five exposures, the corresponding risks were 2.47% and 6.14%, representing absolute risk reductions of 2.66 and 6.18 percentage points, respectively.

| **Risk factor** | **Prevalence, n (%)** | **5-year PAF, % (95% CI)** | **10-year PAF, % (95% CI)** |
| --- | --- | --- | --- |
| Low educational level^a^ | 2,456 (40.81) | 0.04 [0.04,0.04] | 0.04 [0.03,0.04] |
| ***Medication (ATC-code^b^)*** |  |  |  |
| Antihypertensives (C03, C07–C09) | 3,593 (59.70) | 30.09 [29.70,30.43] | 28.71 [28.34,29.06] |
| Lipid-lowering agents (C10) | 2,195 (36.47) | 26.65 [26.02,27.27] | 25.17 [24.58,25.73] |
| Antidepressants (N06A) | 1,087 (18.06) | 6.98 [6.61,7.35] | 6.52 [6.17,6.87] |
| Prednisolone (H02AB06) | 495 (8.23) | 1.34 [1.22,1.46] | 1.22 [1.14,1.37] |
| **Combined PAF** |  | **51.82 [51.22,52.42]** | **50.19 [49.57,50.73]** |

**Panel B. Control cohort**
The adjusted observed cumulative risk was 6.28% at 5 years and 15.58% at 10 years. Under the joint counterfactual elimination of all five exposures, the corresponding risks were 3.20% and 8.25%, representing absolute risk reductions of 3.08 and 7.33 percentage points, respectively.

| **Risk factor** | **Prevalence, n (%)** | **5-year PAF, % (95% CI)** | **10-year PAF, % (95% CI)** |
| --- | --- | --- | --- |
| Low educational level^a^ | 23,031 (40.02) | 8.76 [8.67,8.84] | 8.15 [8.07,8.23] |
| ***Medication (ATC-code^b^)*** |  |  |  |
| Antihypertensives (C03, C07–C09) | 31,032 (53.93) | 34.90 [34.72,35.04] | 33.02 [32.87,33.17] |
| Lipid-lowering agents (C10) | 17,081 (29.68) | 10.50 [10.39,10.61] | 9.59 [9.49,9.68] |
| Antidepressants (N06A) | 10,717 (18.62) | 2.40 [2.35,2.45] | 2.22 [2.18,2.25] |
| Prednisolone (H02AB06) | 7,209 (12.53) | 3.47 [3.39,3.55] | 3.16 [3.09,3.23] |
| **Combined PAF** |  | **49.05 [48.87,49.22]** | **47.07 [46.88,47.22]** |

**Panel C. Total cohort**

The adjusted observed cumulative risk was 6.17% at 5 years and 15.27% at 10 years. Under the joint counterfactual elimination of all five exposures, the corresponding risks were 3.14% and 8.07%, representing absolute risk reductions of 3.03 and 7.20 percentage points, respectively.

| **Risk factor** | **Prevalence, n (%)** | **5-year PAF, % (95% CI)** | **10-year PAF, % (95% CI)** |
| --- | --- | --- | --- |
| Low educational level^a^ | 25,487 (40.10) | 8.07 [8.00,8.14] | 7.52 [7.45,7.59] |
| ***Medication (ATC-code^b^)*** |  |  |  |
| Antihypertensives (C03, C07–C09) | 34,625 (54.48) | 34.59 [34.45,34.73] | 32.75 [32.60,32.93] |
| Lipid-lowering agents (C10) | 19,276 (30.33) | 11.64 [11.52,11.75] | 10.66 [10.56,10.77] |
| Antidepressants (N06A) | 11,804 (18.57) | 2.73 [2.67,2.77] | 2.52 [2.48,2.57] |
| Prednisolone (H02AB06) | 7,704 (12.12) | 3.26 [3.19,3.33] | 2.98 [2.92,3.04] |
| **Combined PAF** |  | **49.09 [48.92,49.26]** | **47.14 [46.98,47.33]** |

*^a^ Low educational level defined as no higher than 2 years of upper secondary school.*

*^b^ ATC = Anatomical Therapeutic Chemical Classification System*
